# Antiseizure Effects of Cannabidiol Leading to Increased Peroxisome Proliferator-Activated Receptor Gamma Levels in the Hippocampal CA3 Subfield of Epileptic Rats

**DOI:** 10.3390/ph15050495

**Published:** 2022-04-19

**Authors:** Anna-Maria Costa, Fabiana Russo, Lara Senn, Davide Ibatici, Giuseppe Cannazza, Giuseppe Biagini

**Affiliations:** 1 Department of Biomedical, Metabolic and Neural Sciences, University of Modena and Reggio Emilia, 287 Campi Street, 41125 Modena, Italy; annamaria.costa@unimore.it (A.-M.C.); lara.senn@unimore.it (L.S.); 226817@studenti.unimore.it (D.I.); 2 Ph.D. School of Clinical and Experimental Medicine (CEM), University of Modena and Reggio Emilia, 287 Campi Street, 41125 Modena, Italy; fabiana.russo@unimore.it; 3 Department of Life Sciences, University of Modena and Reggio Emilia, 103 Campi Street, 41125 Modena, Italy; giuseppe.cannazza@unimore.it

**Keywords:** cannabinoids, cannabidiol, drug-resistant epilepsy, kainic acid, nuclear receptors, peroxisome proliferator-activated receptor gamma, temporal lobe epilepsy

## Abstract

We evaluated the effects of cannabidiol (CBD) on seizures and peroxisome proliferator-activated receptor gamma (PPARγ) levels in an animal model of temporal lobe epilepsy (TLE). Adult male Sprague-Dawley rats were continuously monitored by video-electrocorticography up to 10 weeks after an intraperitoneal kainic acid (15 mg/kg) injection. Sixty-seven days after the induction of *status epilepticus* and the appearance of spontaneous recurrent seizures in all rats, CBD was dissolved in medium-chain triglyceride (MCT) oil and administered subcutaneously at 120 mg/kg (n = 10) or 12 mg/kg (n = 10), twice a day for three days. Similarly, the vehicle was administered to ten epileptic rats. Brain levels of PPARγ immunoreactivity were compared to those of six healthy controls. CBD at 120 mg/kg abolished the seizures in 50% of rats (*p* = 0.033 vs. pre-treatment, Fisher’s exact test) and reduced total seizure duration (*p* < 0.05, Tukey Test) and occurrence (*p* < 0.05). PPARγ levels increased with CBD in the hippocampal CA1 subfield and subiculum (*p* < 0.05 vs. controls, Holm–Šidák test), but only the highest dose increased the immunoreactivity in the hippocampal CA3 subfield (*p* < 0.001), perirhinal cortex, and amygdala (*p* < 0.05). Overall, these results suggest that the antiseizure effects of CBD are associated with upregulation of PPARγ in the hippocampal CA3 region.

## 1. Introduction

Epilepsy is a chronic disorder displaying a high impact on psychological, social, and economic conditions of patients and their caregivers, besides the problems of the disease itself [1]. Its prevalence of 0.5% to 1.0% within the world’s population ranks epilepsy on the fourth place of neurological diseases after migraine-headache, stroke, and dementia [2]. Among others, temporal lobe epilepsy (TLE) is the most common form of epilepsy in adult patients [3]. The administration of antiseizure medications (ASMs) results in seizure freedom in about 65% of patients [4]. However, the remaining patients continue to present spontaneous recurrent seizures (SRSs). For this reason, the use of alternative drugs as new treatments is in the focus of the latest research.

Cannabidiol (CBD) and tetrahydrocannabinol (∆^9^-THC) are the two major active substances found in the female flowers of *Cannabis sativa* L. Due to the lack of psychotropic properties of CBD, which are the principal drawbacks of ∆^9^-THC, its application is declared to be more secure and effective in reducing seizures. Therefore, in the last five decades, CBD has gained considerable interest as an add-on medication for drug-resistant epilepsy. In 2018, CBD was approved by the Food and Drug Administration (FDA) for the treatment of severe forms of infantile epilepsy, such as Lennox–Gastaut and Dravet syndromes [5,6].

The medicinal value of CBD has been recognized for many years [7], nevertheless, significant progress in establishing the clinical protocol to use CBD as an add-on ASM has only been set in the recent period. Although the exact mechanism behind the functioning of CBD is not fully confirmed yet, there are several targets exhibiting promising results in their mutual dependence and interaction [8,9,10]. For example, CBD has been shown to act as a reuptake inhibitor of the endocannabinoid anandamide, changing the excitatory and inhibitory dynamics of synapses [11]. In addition to this, it has been demonstrated that the PI3K/mTOR pathway, which is linked to numerous neurological disorders, including epilepsy, is involved in the anticonvulsant effects of CBD [12]. Moreover, CBD inhibits adenosine transport type 1 equilibrative nucleoside transporter (ENT-1) activity and lowers neuronal excitability by functionally antagonizing G-protein-coupled receptor 55 (GPR55) and desensitizing transient receptor potential cation channel subfamily V member 1 (TRPV1) receptors [13]. 

Another beneficial interaction was found between peroxisome proliferator-activated receptor gamma (PPARγ) and CBD, which was associated with neuroprotective properties in ischemic stroke and with stimulated neurogenesis in a model of Alzheimer’s disease [9,14,15]. CBD has already been shown to regulate immune responses by modulating the nuclear factor kappa-light-chain-enhancer of activated B cells (NF-κB) signaling, a pathway activated in both reactive microglia and astrocytes and hypothesized to contribute to neurodegenerative diseases such as Parkinson’s disease. Notably, PPARγ activation inhibits NF-κB signaling and lowers the mRNA levels of proinflammatory mediators including tumor necrosis factor alpha, interleukin 6 (IL-6), and inducible nitric oxide synthase [16,17]. The ability of glial cells to promote the inflammatory state by producing inflammatory mediators during an epileptic seizure emphasizes the neuroprotective role of CBD, possibly mediated by PPARγ, in epilepsy [18]. However, the fact that CBD presents agonist, antagonist and other modulatory actions on diverse receptors and channels, complicates the determination of its exact pathway [8].

In preclinical studies, it was suggested that CBD attenuates seizures, improves survival, and behavioral comorbidities of Dravet syndrome in mice [19,20]. Similarly, CBD exerts anticonvulsant activities in a wide variety of acute seizure models, such as the audiogenic seizure, maximal electroshock, and 6 Hz-seizure models [21,22], or other models in which seizures are induced by isoniazid, cocaine, or gamma-aminobutyric acid type A (GABA_A_) receptor antagonists [23,24,25]. Additionally, CBD seems to display anti-ictal activities in rodents when administered prior to the pilocarpine or penicillin-induced seizures [26,27]. It can also decrease the percentage of rats developing *status epilepticus* (SE) after the intrahippocampal administration of pilocarpine [28]. Then, antiepileptogenic effects of CBD have been reported in a rat model of pentylenetetrazole (PTZ)-induced kindling [29], whereas anticonvulsant effects against the motor manifestations of a focal epilepsy have been described in cobalt-treated rats [30]. Furthermore, chronic CBD administration was shown to ameliorate both reference and working memory errors after the onset of SRSs, in a model of TLE [27].

Considering the above-mentioned data, it could be possible that the antiepileptic effects of CBD depend, at least in part, on unconventional targets [9], such as PPARγ. In this regard, we recently found that PPARγ is involved in the antiseizure properties of EP-80317, a ghrelin analogue tested in a model of repeated seizure induction [31]. Thus, to evaluate this hypothesis, we designed an experiment in which two different doses of CBD (12 or 120 mg/kg) [21,32,33,34] were tested in epileptic rats previously treated with kainic acid (KA) [35,36]. In these animals, we evaluated the changes in levels of PPARγ immunoreactivity in response to the antiseizure effects of CBD.

## 2. Results

### 2.1. Different Responses to Treatment

**Figure 1:**
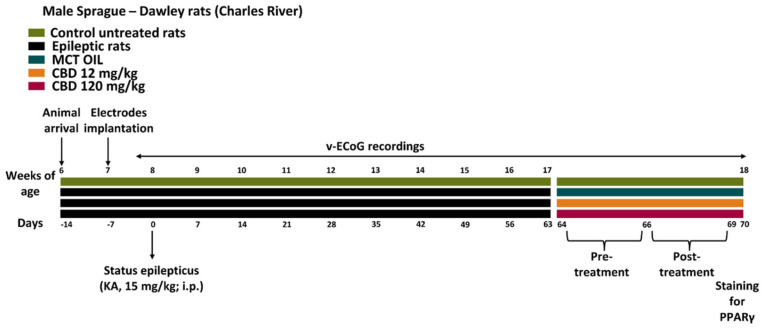
Experimental design. Thirty-eight male Sprague-Dawley rats were purchased at 6 weeks of age, and cortical electrodes were implanted 1 week later for video-electrocorticographic (v-ECoG) recordings of recurrent seizures. *Status epilepticus* (SE) was induced with an intraperitoneal (i.p.) administration of kainic acid (KA; 15 mg/Kg) at 8 weeks of age (n = 2 rats died during the SE). Ten epileptic rats per group were treated with cannabidiol (CBD) at 12 mg/kg, 120 mg/kg, or with medium-chain triglyceride (MCT) oil. The remaining animals (n = 6) were used as control, non-treated rats.

Thirty epileptic rats were obtained by injecting KA in 32 animals (mortality, approximately 7%), as illustrated in Figure 1, and monitoring the epileptic activity by continuous video-electrocorticographic (v-ECoG) recordings until treatment with CBD or its vehicle. Further details about the experimental design are available in the method section.

During the treatment with CBD (120 mg/kg dissolved in medium-chain triglyceride (MCT) oil, injected subcutaneously, s.c.), 50% of the rats presented with a complete response, consisting of the suppression of SRSs during the overall period of treatment. The remaining rats experienced an incomplete response to CBD, as the seizure suppression was not complete but only reduced to 63%. By comparing the occurrence of SRSs under the administration of a high dose of CBD with that observed in the pre-treatment period (during which all of the rats displayed at least one SRS) of the same animals, we observed a significant difference (*p* = 0.033, Fisher’s exact test), suggesting that the high dose of CBD efficaciously suppressed ictogenesis. In comparison to the pre-treatment period, the treatment with a low dose of CBD or MCT oil did not affect the number of rats that displayed seizures, as only 2 out of 10 rats did not present SRSs after the treatment with CBD at 12 mg/kg, and only 1 out of 10 rats did not develop SRSs during the administration of MCT oil.

### 2.2. Characterization of the Duration of SRSs

**Figure 2:**
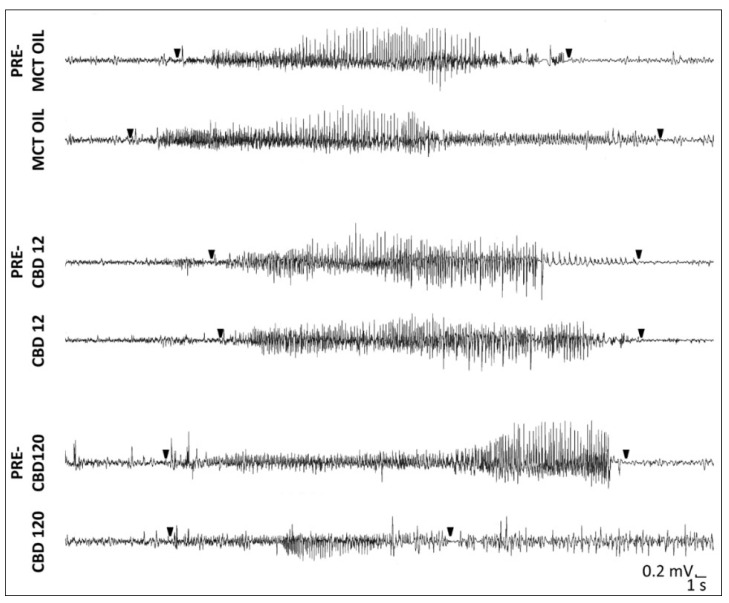
Spontaneous recurrent seizures (SRSs) after induction of *status epilepticus* in epileptic rats treated with cannabidiol (CBD) at 12 mg/kg, 120 mg/kg, or with medium-chain triglyceride (MCT) oil. SRSs were recorded in rats during the treatment with CBD or MCT oil, but also 3 days before (PRE-MCT OIL, PRE-CBD12, and PRE-CBD120) the treatment. Notably, SRSs during the high concentrations of CBD belong to incomplete responders. Arrowheads represent the onset and termination of SRSs.

By visual inspection, SRSs recorded in CBD-treated rats (120 mg/kg), which did not completely respond to CBD administration, differed in duration when compared to the SRSs observed in the same group of rats during the days preceding the treatment, and from those characterized in epileptic rats treated with 12 mg/kg of CBD or MCT in the same period (Figure 2). 

**Figure 3:**
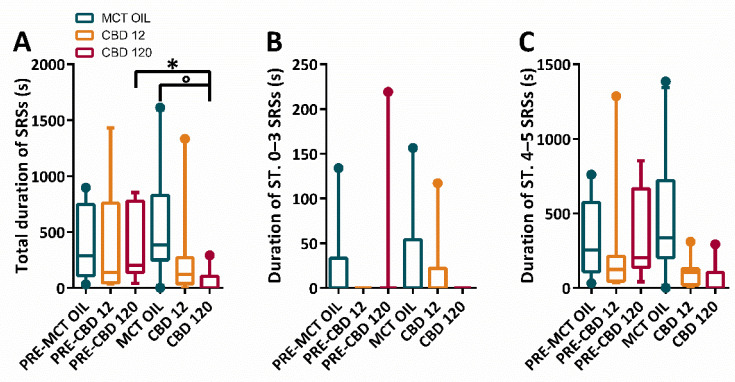
Duration of spontaneous recurrent seizures (SRSs) in epileptic rats treated with cannabidiol (CBD) at 12 mg/kg, 120 mg/kg or with medium-chain triglyceride (MCT) oil. Rats (n = 30) were treated with CBD 12 mg/kg (n = 10, 2 of which did not display SRSs and were attributed a 0 value), CBD 120 mg/kg (n = 10, 5 of which did not display SRSs and were attributed a 0 value), or MCT oil (n = 10, 1 of which did not display SRSs and were attributed a 0 value). In (**A**), CBD at 120 mg/kg significantly decreased (Tukey test; * *p* < 0.05, PRE-CBD 120 vs. CBD 120; ° *p* < 0.05, MCT OIL vs. CBD 120) the total number of SRSs. The high dose of CBD displayed no significant effects when stages (ST.) 0–3 (**B**) and ST. 4–5 (**C**) SRSs were considered separately.

The changes in the duration of SRSs with different treatment conditions were evaluated through the Friedman repeated measures analysis of variance (ANOVA) on Ranks. This analysis revealed that differences in the median duration of total SRSs were significantly different across treatment groups (*p* = 0.026). The Tukey test showed that the treatment with high dose of CBD induced a significant decrease in the total duration of all SRSs (*p* < 0.05), in comparison to the pre-treatment period. In the same period, a significant difference was also observed between the treatment with a high dose of CBD and MCT oil (*p* < 0.05). Interestingly, these changes were not significantly different when stages (ST.) 0–3 and ST. 4–5 SRSs were considered separately (Figure 3A–C).

### 2.3. Characterization of the Total Number of SRSs

In Table 1, the total number of SRSs with different treatment conditions was reported. 

**Figure 4:**
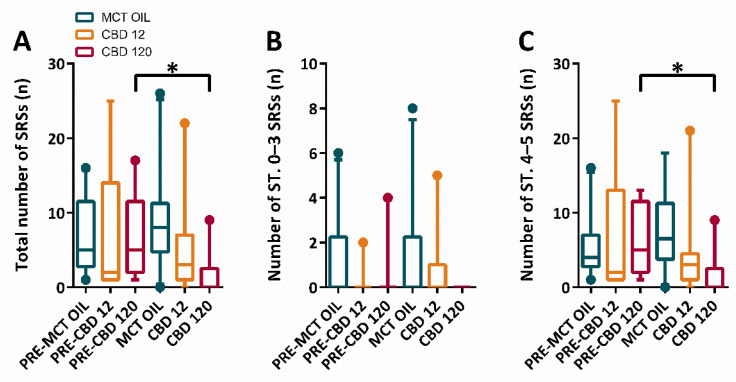
Total number of spontaneous recurrent seizures (SRSs) in epileptic rats treated with cannabidiol (CBD) at 12 mg/kg, 120 mg/kg, or with medium-chain triglyceride (MCT) oil. Rats (n = 30) were treated with CBD 12 mg/kg (n = 10, 2 of which did not display SRSs and were attributed a 0 value), CBD 120 mg/kg (n = 10, 5 of which did not display SRSs and were attributed a 0 value), or MCT oil (n = 10, 1 of which did not display SRSs and were attributed a 0 value). In (**A**), CBD at 120 mg/kg significantly decreased (Tukey test; * *p* < 0.05, PRE-CBD 120 vs. CBD 120) the total number of SRSs. The high dose of CBD displayed no significant effects on stages (ST.) 0–3 SRSs (**B**), but only on ST. 4–5 SRSs (**C**).

Then, the changes in the occurrence of SRSs were evaluated through the Friedman repeated measures ANOVA on Ranks. This analysis revealed that differences in the median number of total SRSs were significantly different across treatment groups (*p* = 0.035). The Tukey test (Figure 4A–C) showed that the treatment with high dose of CBD induced a significant decrease in the total number of all SRSs (*p* < 0.05), in comparison to the pre-treatment period. Interestingly, the effects of CBD on the median number of SRSs were limited to tonic-clonic SRSs (*p* < 0.05).

### 2.4. PPAR-γ Immunoreactivity

**Figure 5:**
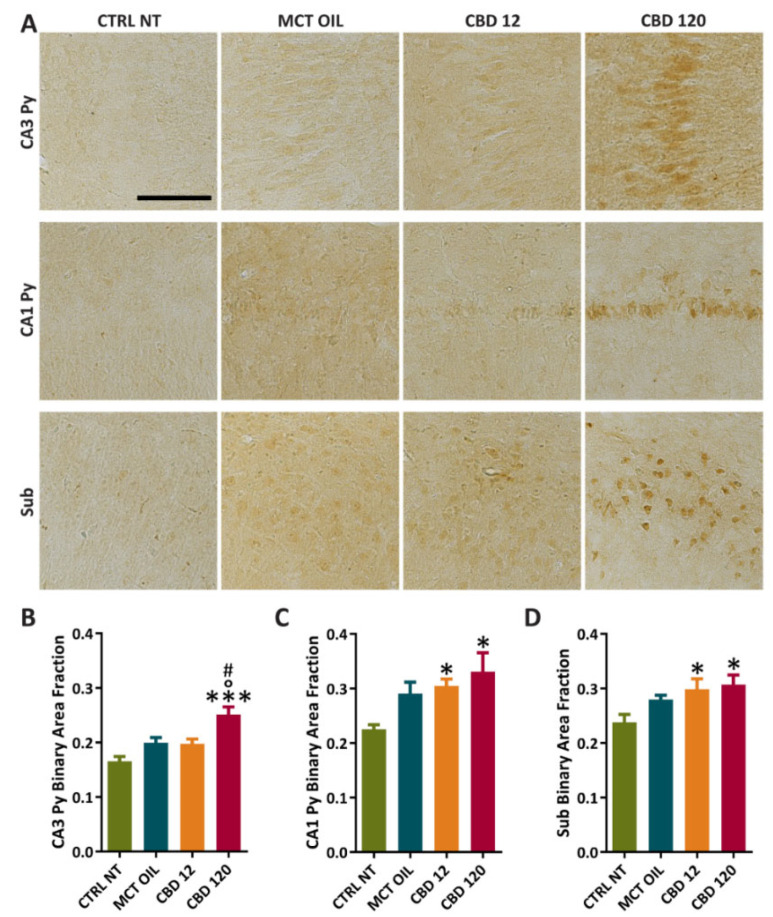
PPARγ immunoreactivity in hippocampal regions of controls and rats treated with cannabidiol (CBD) or with medium-chain triglyceride (MCT) oil. Non-treated controls (CTRL NT, n = 6) and epileptic animals (**A**) treated with CBD at high dose (CBD 120, n = 6), CBD at low dose (CBD 12, n = 6), or MCT oil (MCT OIL, n = 6) were used to determine the levels of PPARγ in CA3 Py (**B**), CA1 Py (**C**), and Sub (**D**). * *p* < 0.05, *** *p* < 0.001, CBD 12 or CBD120 vs. CTRL NT; ° *p* < 0.05, CBD 120 vs. MCT OIL; # *p* < 0.05, CBD 120 vs. CBD 12; Holm-Šidák test. CTRL NT, non-treated control; CBD, cannabidiol. CA3 Py, *Stratum pyramidalis of Cornu Ammonis 3*; CA1 Py, *Stratum pyramidalis of Cornu Ammonis 1*; Sub, Subiculum. Scale: 0.1 mm.

We evaluated the effects of treatment with CBD or MCT oil on PPARγ immunoreactivity (Figure 5A–D) in the *Cornu Ammonis 3 stratum pyramidalis* (CA3 Py), *Cornu Ammonis 1 stratum pyramidalis* (CA1 Py), and subiculum (Sub). Note that six healthy control rats and six rats per treatment group were randomly chosen for the immunohistochemical staining, whereas 4 out of 10 epileptic rats per treatment group were monitored by video-electrocorticography for an additional week to assess possible changes in seizure frequency after treatment. However, no remarkable changes in seizure frequency were visually observed in the week following the treatment.

In comparison to controls, the treatment with CBD at 120 mg/kg significantly increased PPARγ levels in CA3 Py (0.166 ± 0.009 vs. 0.251 ± 0.014, *p* < 0.001; Holm–Šidák test), CA1 Py (0.225 ± 0.009 vs. 0.331 ± 0.035, *p* = 0.01), and Sub (0.238 ± 0.014 vs. 0.307 ± 0.018, *p* = 0.024). Furthermore, PPARγ levels in CA3 Py were significantly increased after the treatment with a high dose of CBD, in comparison to the groups treated with CBD at 12 mg/kg (0.198 ± 0.008 vs. 0.251 ± 0.014, *p* = 0.013) or MCT (0.200 ± 0.009 vs. 0.251 ± 0.014, *p* = 0.014). In comparison to controls, PPARγ levels were significantly higher in CA1 Py (0.225 ± 0.009 vs. 0.305 ± 0.013, *p* = 0.04) and Sub (0.238 ± 0.014 vs. 0.299 ± 0.019, *p* = 0.045) after the treatment with CBD at 12 mg/kg.

**Figure 6:**
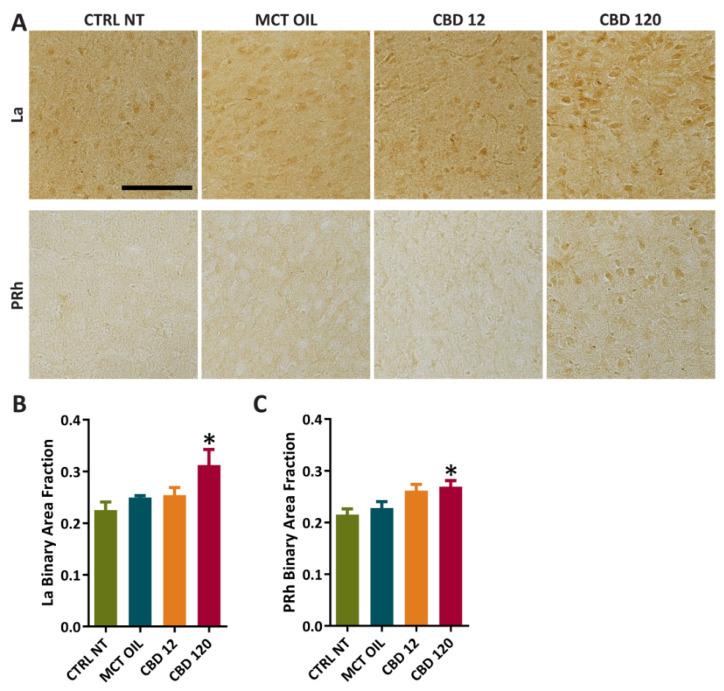
PPARγ immunoreactivity in extrahippocampal regions of controls and rats treated with cannabidiol (CBD) or with medium-chain triglyceride (MCT) oil. Non-treated controls (CTRL NT, n = 6) and epileptic animals (**A**) treated with CBD at high dose (CBD 120, n = 6), CBD at low dose (CBD 12, n = 6), or MCT oil (MCT OIL, n = 6) were used to determine the levels of PPARγ in lateral amygdala (La) (**B**), and perirhinal cortex (PRh) (**C**). * *p* < 0.05, CBD 120 vs. CTRL NT; Holm-Šidák test. Scale: 0.1 mm.

Furthermore, we assessed the effects of treatment with CBD or MCT oil on PPARγ immunoreactivity (Figure 6A–C) in the lateral amygdala (La) and perirhinal cortex (PRh). In comparison to controls, the treatment with CBD at 120 mg/kg significantly increased PPARγ levels in La (0.225 ± 0.015 vs. 0.312 ± 0.030, *p* = 0.012) and PRh (0.215 ± 0.011 vs. 0.269 ± 0.012, *p* = 0.045).

## 3. Discussion

The present study aimed to evaluate the efficacy of CBD in an animal model of TLE and to analyze the modulation of PPARγ immunoreactivity by CBD. Particularly, the subcutaneous injections of CBD at 120 mg/kg reduced: (i) the seizure duration; (ii) the number of rats experiencing SRSs; (iii) the total number of SRSs in epileptic rats. Moreover, PPARγ immunoreactivity was increased in hippocampal and extrahippocampal regions after the repeated administration of CBD, with a remarkable effect of the higher dose in the CA3 hippocampal subfield.

A compulsory step to assess the efficacy of CBD in controlling SRSs was to define its therapeutic dose. To this aim, we adopted the intraperitoneal (i.p.) KA model [35,36], and CBD was administered at 12 or 120 mg/kg. In our experiment, only the highest dose displayed a therapeutic effect in epileptic rats. Thus, this finding confirms the results of other models. For instance, the i.p. injection of CBD at 25 mg/kg did not change the seizure thresholds in the maximal electroshock seizure threshold test and the 6-Hz-induced seizure test, whereas its injection at higher doses was significantly more powerful in counteracting seizures [21]. Indeed, only CBD at 100 mg/kg significantly potentiated the anticonvulsant effect of topiramate, oxcarbazepine, and pregabalin in the maximal electroshock seizure threshold test. Similarly, only CBD at 50 and 100 mg/kg enhanced the activity of tiagabine and gabapentin in the 6 Hz seizure test [21]. This agrees with recent data, which demonstrated that the administration of CBD at 25 mg/kg by gavage was ineffective at reducing seizures induced by the repeated 6-Hz corneal stimulation test in mice. However, a more complete whole-plant extract (i.e., oil enriched with terpenes) was more effective and reduced the therapeutic dose [22]. 

An important issue to consider when adopting CBD to control SRSs might be that the mechanisms underlying the anticonvulsant activity of CBD are not completely defined [8,9]. CBD could mediate the hyperpolarization of presynaptic membrane and reduce the neuronal hyperactivity through an indirect action on CB1 receptors, as it seemed to act as a reuptake inhibitor of the endocannabinoid anandamide [37]. However, the involvement of additional targets could further explain the antiseizure properties of CBD in our animal model of TLE. For instance, CBD induced neuroprotection in ischemic stroke though PPARγ involvement [14], which seemed to be also related to the antiseizure properties of the ghrelin receptor antagonist EP-80317 [31], and of the ketogenic diet [38]. Moreover, CBD might exert anti-inflammatory effects by acting both on the peripheral system and central nervous system. More precisely, CBD seemed to reduce peripheral inflammation by acting at TRPV1, cannabinoid receptor 2 (CB2), and GPR55 receptors. These interactions were, in turn, involved in the downregulation of enzymes related to the production of prostaglandins, reactive oxygen species, and cytokines. At variance, the anti-inflammatory effects of CBD in the central nervous system seemed to involve the inhibition of the mitogen-activated protein kinase pathway and the downregulation of NF-κB, together with PPARγ-mediated reduction of lipid peroxidation [7]. In this regard, the possible involvement of PPARγ in the neuroprotective and anti-inflammatory effects of CBD could be further supported by the fact that also the PPARγ-agonist pioglitazone was shown to reduce neuronal loss and decrease the levels of IL-1β and IL-6 in the hippocampus of a rat model of PTZ induced SE [39]. Additionally, pioglitazone was shown to attenuate pilocarpine-induced seizure severity, neuronal loss, blood-brain-barrier impairment, and sodium currents in hippocampal neurons. Neuronal excitability and excitotoxicity, in contrast, were exacerbated by PPARγ inhibition [40].

In general, the complete understanding of the signaling pathways involved in the beneficial effects of CBD could help in defining the efficacy and safety of CBD as an add-on anticonvulsant therapy in different populations of epileptic patients [41], so not only in those populations in which CBD was already shown to display beneficial effects (e.g., patients with treatment-resistant Lennox–Gastaut syndrome and Dravet syndrome) [5,6]. In our experiment, data suggested that the levels of immunoreactivity for PPARγ were significantly increased within brain regions in a dose-related manner, and the CA3 hippocampal subfield emerged as the most important target region for the modulation of PPARγ levels by the effective CBD dose. Overall, these results might contribute to explaining, at least in part, the antiseizure properties of CBD in our animal model of TLE.

## 4. Materials and Methods

### 4.1. Animals

The study protocol was authorized by the Italian Ministry of Health (729/2021-PR), after approval by the university Animal Welfare Body. All experiments were performed in agreement with the European Directive 2010/63/EU and the consequent Italian act (DM 26/2014). Thirty-eight adult male Sprague-Dawley rats (Charles River, Calco, Italy), with initial weights of 175–200 g, were housed in a specific pathogen-free facility in a controlled environment and with ad libitum access to water and food. All efforts were made to refine procedures and protect the animals’ welfare.

### 4.2. Experimental Design

SE was induced by an i.p. injection of KA (15 mg/kg; Sigma-Aldrich, Milan, Italy). At the end of SE, softened rat chow was administered to minimize discomfort. The mortality rate in our systemic KA rat model was similar to that previously determined [35]. In ten epileptic rats per group, both CBD (12 or 120 mg/kg; provided by Farmabios, Gropello Cairoli, Italy) and MCT oil (1 mL/kg; USP pharmaceutical grade MCT Lean; MCT Foods, Glencoe, IL, USA) were administered s.c. twice a day for three days, starting from 67 days after SE onset. Particularly, the subcutaneous administration was preferred because substances might be absorbed at a slower rate compared with other parenteral routes, providing a sustained effect [42]. Moreover, the injection was performed twice a day to mimic the administration of Epidiolex^®^ in patients affected by drug-resistant SRSs [5]. For immunohistochemical analyses, six rats per treatment group (n = 18) and six untreated controls were considered. Specifically, the control rats were used to determine the basal levels of PPARγ.

### 4.3. Electrode Implantation and Video-Electrocorticography

Guiding holes were drilled and electrodes (stainless steel Ø = 1 mm; PlasticsOne, Roanoke, VA, USA) were implanted in frontal (bregma 0 mm, 3.5 mm lateral from midline) and occipital cortices (bregma −6.5 mm, 3.5 mm lateral from midline) of both hemispheres. One additional electrode was implanted below the lambda on the midline in all rats and used as a reference. During electrode implantation, volatile isoflurane was used to induce a deep anesthesia, assessed by deep breath, loss of tail and eye reflexes. Gel containing 2.5 g lidocaine chloride, 0.5 g neomycin sulfate, and 0.025 g fluocinolone acetonide (Neuflan^®^ gel; Molteni Farmaceutici, Scandicci, Italy) was applied at the end of the surgical procedure to reduce acute pain and risk of infection. All animals were monitored until complete recovery from anesthesia and housed in single cages with no grids or environmental enrichments to avoid risk of headset loss. 

The EcoG data were recorded via cable connection between headset and preamplifiers. Electrical activity was digitally filtered (0.3 Hz high-pass, 500 Hz low-pass), and acquired at 1 kHz per channel. All data was stored on a personal computer after the mathematical subtraction of traces of recording electrodes from trace of reference electrode by using a PowerLab8/30 amplifier connected to 4 BioAmp preamplifiers (ADInstruments; Dunedin, Otago, New Zealand). Videos were digitally captured through a camera connected to the dedicated computer and synchronized to the ECoG traces through LabChart 8 PRO internal trigger.

### 4.4. Behavioral and v-ECoG Analysis

ECoG traces were digitally filtered offline (band-pass: high 50 Hz, low 1 Hz) and manually analyzed using LabChart 8 PRO software (AD Instruments, Sydney, Australia) by expert raters. SE was defined as the period of time in which rats either did not recover normal behavior between a seizure and the other, or in which they displayed continuous shaking for more than 5 min. The end of SE was defined by a progressive reduction in frequency of the continuous electrographic spikes, accompanied by the recovery of normal behavior. In our animal model, SE was allowed to self-terminate, and the mean duration was about 10 h [35]. 

Seizures were defined as ECoG segments with a minimum duration of 10 s, continuous synchronous high-frequency activity, and an amplitude of at least twice the previous baseline. The mean duration of SRSs per day was equal to 0 s when the animal did not display SRSs for at least 24 h. Seizures and their durations were determined in the ECoG traces, then investigated for correlated behavior by using the synchronized video recordings. In particular, all seizures were scored as ST. 0 (or subclinical) if a clear epileptiform ECoG signal was observed without corresponding evident behavior in the video; ST. 1–2 in the presence of absence-like immobility, “wet-dog shakes”, facial automatisms, and head nodding; ST. 3, when presenting with forelimb clonus and lordosis posture; ST. 4, corresponding to generalized seizures and rearing; ST. 5, when seizures consisted of rearing with loss of posture and/or wild running, followed by generalized convulsions.

### 4.5. Immunohistochemistry

Rats deeply anesthetized with isoflurane were transcardially perfused with phosphate buffered saline (PBS, pH 7.4) followed by Zamboni’s fixative (pH 6.9), 12 h after the last subcutaneous injection of CBD or MCT oil. Brains were post-fixed at 4 °C in the same fixative for 24 h, cryoprotected in 15 and 30% sucrose solutions, and stored at −80 °C until used. Horizontal sections of 50 µm were cut using a freezing sliding microtome (Leica SM2000 R; Leica, Nussloch, Germany). Brain sections were washed in Tris-buffered saline (TBS) and incubated in 3% H_2_O_2_ in TBS (30 min). After washing, sections were blocked 1 h in TBS containing 2% bovine serum albumin, 0.3% Triton X-100 (Tx) and 5% normal goat serum. Brain sections were then placed at 4 °C with a polyclonal rabbit anti-PPARγ (Ab209350; Abcam, Cambridge, UK, dilution 1:2000, 48 h). Following another washing step, sections were incubated for 1 h with a biotinylated anti-rabbit secondary antibody (Vector Laboratories, Burlingame, CA, USA; 1:200), and later with the avidin-biotin-peroxidase complex (Elite ABC Kit; Vector Laboratories, Burlingame, CA, USA). The staining was performed in 0.05% 3,3-diaminobenzidine tetrahydrochloride for 5 min (DAB, Sigma–Aldrich, Milan, Italy) and developed by adding 0.03% H_2_O_2_. In the end, sections were washed again in TBS, mounted on gelatin-coated slides and cover slipped with Eukitt (Eukitt R, O. Kindler GmbH & Co., Freiburg, Germany).

### 4.6. Image Analysis

The levels of immunoreactivity for PPARγ were in the CA3 Py, CA1 Py, Sub, La, and in the PRh. The staining was performed as previously detailed [31]. Immunostained sections from −8.04 mm to −5.04 mm Bregma level were evaluated with a Nikon Eclipse CiL (Nikon Instruments) at 20X, and images were digitally captured by a Nikon DS-Fi3 digital camera. PPARγ immunoreactivity was measured as a binary area fraction after discrimination from background staining. Particularly, the binary area fraction (binary area/measured area) was analyzed using NIS-Elements software. Particularly, the binary area represented the sum of areas of all binary objects, whereas the measured area was the region of interest (ROI) within the measurement frame. In all sections, the ROI was manually traced.

### 4.7. Statistical Analysis

For all groups, we compared the number of rats with SRSs before and during the treatment period by using the Fisher’s exact test. Data on the total number and duration of SRSs were analyzed by Friedman repeated measures ANOVA on Ranks, followed by the Tukey test. PPARγ levels were compared using one-way ANOVA, followed by Holm–Šidák test. One outlier per data set was identified using the Grubbs’ test and removed. Statistical analyses were performed by using SigmaPlot 13 (Systat Software; San Jose, CA, USA). The results are shown as medians and interquartile ranges (seizures) or mean ± standard error of the mean (SEM) (PPARγ) and considered significant at *p* < 0.05.

## 5. Conclusions

The present work was aimed at evaluating the effects of CBD on SRSs and PPARγ levels in an animal model of TLE. 

The results obtained on the systemic KA rat model suggested that the antiseizure effects of CBD might lead, at least in part, to increased PPARγ levels in hippocampal and extrahippocampal regions, and especially in the hippocampal CA3 subfield.

A possible limitation suggested in this study is the lack of definitive proof of the involvement of PPARγ in the antiseizure effects of CBD. In the future, we will assess whether PPARγ antagonists block the antiseizure effects of CBD. 

## Figures and Tables

**Table 1:** Median and interquartile range values of the number of spontaneous recurrent seizures (SRSs) in the various treatment groups with cannabidiol (CBD) at 12 mg/kg, 120 mg/kg, or with medium-chain triglyceride (MCT) oil, subdivided by stages (ST.), or considered altogether. Ten animals per group were included, except where a single outlier per group was identified and removed.

Total Number of SRSs			
Group	Pre-Treatment	Treatment	Outlier Removal
MCT OIL	5.00 (2.75–11.50)	8.00 (4.75–11.25)	no
CBD 12	2.00 (1.00–14.00)	3.00 (1.00–7.00)	yes
CBD 120	5.00 (2.00–11.50)	0.00 (0.00–2.50)	yes
Number of ST. 0–3 SRSs			
MCT OIL	0.00 (0.00–2.25)	0.00 (0.00 – 2.25)	no
CBD 12	0.00 (0.00–0.00)	0.00 (0.00–1.00)	yes
CBD 120	0.00 (0.00–0.00)	0.00 (0.00–1.00)	yes
Number of ST. 4–5 SRSs			
MCT OIL	4.00 (2.75–7.00)	6.50 (3.75–11.25)	no
CBD 12	2.00 (1.00–13.00)	3.00 (1.00–4.50)	yes
CBD 120	5.00 (2.00–11.50)	0.00 (0.00–2.50)	yes

## Data Availability

The data is contained within article.
